# Federated Learning for 5G Radio Spectrum Sensing

**DOI:** 10.3390/s22010198

**Published:** 2021-12-28

**Authors:** Małgorzata Wasilewska, Hanna Bogucka, Adrian Kliks

**Affiliations:** Institute of Radiocommunications, Poznan University of Technology, 61-131 Poznań, Poland; hanna.bogucka@put.poznan.pl (H.B.); adrian.kliks@put.poznan.pl (A.K.)

**Keywords:** spectrum sensing, machine learning, 5G, LTE, federated learning, convolutional neural network, deep learning, clustering, cognitive radio

## Abstract

Spectrum sensing (SS) is an important tool in finding new opportunities for spectrum sharing. The users, called Secondary Users (SU), who do not have a license to transmit without hindrance, need to employ SS in order to detect and use the spectrum without interfering with the licensed users’ (primary users’ (PUs’)) transmission. Deep learning (DL) has proven to be a good choice as an intelligent SS algorithm that considers radio environmental factors in the decision-making process. It is impossible though for SU to collect the required data and train complex DL models. In this paper, we propose to employ a Federated Learning (FL) algorithm in order to distribute data collection and model training processes over many devices. The proposed method categorizes FL devices into groups by their mean Signal-to-Noise ratio (SNR) and creates a common DL model for each group in the iterative process. The results show that detection accuracy obtained via the FL algorithm is similar to detection accuracy obtained by employing several DL models, namely convolutional neural networks (CNNs), specialized in spectrum detection for a PU signal with a given mean SNR value. At the same time, the main goal of simplification of the SS process in the network is achieved.

## 1. Introduction

In today’s wireless communication systems, the radio spectrum has become a scarce resource. For this reason, intelligent spectrum management methods are considered to reuse frequency bands at a certain time and location (so called spectrum gaps or in a more generic sense white spaces) when they are not used by licensed users [1]. In such a scenario, unlicensed users can transmit their signals assuming that the transmission of a licensed user is properly protected. The Spectrum Sensing (SS) methods are supposed to determine whether the licensed user—a primary user (PU)—is transmitting or not, and hence, enable the unlicensed user’s (or a secondary user—SU) transmission. The traditional sensing methods, however, are unable to take full advantage of the time, frequency, or spatial dependencies that exist in detected signals, which results in a rather limited performance of these methods. The machine learning (ML) methods that are able to find intricate features in the input data and recognize present signals are being considered to improve the performance of traditional SS algorithms.

ML algorithms have been widely used for SS applications such as simple spectrum classification methods, for example, in [2,3,4]. ML also proved its usefulness in cooperative sensing, where multiple sensing nodes try to establish spectrum state, e.g., [5,6,7,8]. Here, the learning is applied in order to common information on the spectrum received from multiple sensing devices into one common spectrum decision. ML can be applied for tasks where input data are of a simple form, and the main problem is to find the best decision-making method for the current wireless transmission conditions, as in the aforementioned papers. However, the most valuable use of ML algorithms is when the input data are much more complex and the detection conditions are complicated. In that case, it is important to find a way of finding hidden dependencies between different types of collected data.

A promising approach to improving spectrum sensing efficiency is to predict white spaces based on detected traffic trends and the functioning of the PU. Currently, in most of the telecommunication traffic patterns, intensity fluctuations can be observed that are due to communication demand variations, e.g., daily or weekly variations [9]. Besides the time domain, dependencies and patterns can be observed in frequency and space due to, e.g., applied frequency planning and scheduling, or due to the presence of shadowing occurring in some location areas, etc. Application of ML tools for efficient pattern recognition and white-space detection seems to be a valuable option, as discussed in, e.g., [10,11,12]. In that context, the popular ML algorithms are deep learning (DL) methods, especially recurrent neural networks (RNN), which are known for their abilities to use historical data to make an accurate prediction of the future or present state. The RNN algorithm has been used in [10,11], while [12] focused on support vector regression (SVR)-based algorithm for prediction of PU’s next spectrum state. The frequency patterns combined with time dependencies have been discussed in [13,14,15]. These papers also consider using DL algorithms, namely RNN and convolutional neural network (CNN), which is a great choice for two-dimensional data. The combined approach to signals that depend on time, frequency, and space has been presented in [16], where simple *k*-nearest neighbors (KNN) and random forest (RF) have been used to improve spectrum sensing.

The individual sensing performed by each single SU and employing ML is computationally complex and may be inaccurate. This is because ML algorithms require many training data to be able to recognize the time, frequency, and location dependencies existing in the transmitted and received signal. The end-user terminal usually does not have enough computing and memory resources to store and process the volumes of training data required to train an ML algorithm. Another problem in individual sensing is in obtaining labeled data for supervised learning. In practice, the end user (individual SU) cannot produce their own labeled data, so there appears a need to download them from some external server. This approach is also impractical as it requires communication resources and time to download the data. The introduced delay in downloading the data may cause them to not be useful when a mobile SU changes location in the meantime, which would require retraining the ML model and a new training dataset.

Thus, the stage of creating an ML model should be delegated to more computationally capable (centralized) devices, so that SU could benefit from intelligent sensing methods without the need to spend time, energy, and computational resources on the ML model training. As discussed earlier, a popular idea is to employ cooperative sensing, where SUs exchange their sensing results or collected data to decide cooperatively on the current spectrum state. This approach solves the problem of generating an ML model, which can be created by the elected end user device or the so-called fusion center or a central server, but it still does not answer the problem of collecting labeled data by SUs.

A promising solution to the problems presented above is Federated Learning (FL), which an iterative procedure that edge devices (called FL nodes) that create their ML models on their local data. The created models are then exchanged in a centralized or decentralized manner to create one common ML model that can be shared among mentioned devices. The common model is then adjusted by FL nodes to their local data and the process of local training, exchanging models, and creating the common model is repeated. This method has many advantages. First, it can supply the SU with an ML algorithm suited for its current wireless environment and location, without the need for the SUs to collect data. Second, FL can adapt to the changing radio environment. Finally, SUs need to exchange only a minimal amount of information with the FL server (in the case of centralized FL) or with each other (in the case of decentralized FL) to receive a complete ML model for SS. Whenever SU changes its location or the radio channel quality changes, it receives a new ML model adapted to the channel state.

The FL algorithms have been applied in various contexts related to wireless networks. For example, in [17], the client selection and bandwidth allocation for wireless federated learning networks are discussed, and the authors concentrate on the long-term perspective of resource allocation. An interesting analysis of non-independent and identically distributed (non-IID) data processed in dynamically changing wireless networks is presented in [18], where the averaging scheme is also proposed to reduce the distribution divergence of such kinds of data. Next, in [19], the energy efficiency is discussed in the context of federated learning over wireless networks. A good summary of the challenges and opportunities for wireless federated learning is provided in [20]. Referring to spectrum sensing, in 2019, the authors of [21] proposed the application of FL to spectrum access system (SAS) for the Citizens Broadband Radio Service (CBRS) band system. In particular, by evaluating their non-coherent spectrum-sensing system called FaIR (Federated Incumbent Detection in CBRS), it was shown that FL-based solutions may obtain an improved detection model compared to a naive distributed sensing and centralized model framework. One of the recent papers [22] deals with the introduction of the FL framework for Cooperative Spectrum Sensing (CSS) and proposed FL-based spectrum sensing. Only the two above-mentioned articles relate to SS. To the best of our knowledge, there are no papers that relate to the FL application directly for single-user SS, especially for sensing downlink 5G signals.

Following these observations, in this paper, we propose the application of the centralized FL algorithm for spectrum sensing by improving spectrum usage prediction. The dedicated SS sensor devices are used as FL nodes, which are responsible for collecting sensing data, creating local ML models, and exchanging those models with the FL server. In the first step, we apply SS node clustering based on the observed Signal-to-Noise ratio (SNR) as their inputs, to identify FL nodes, which should share the same spectrum occupation model. As a shared ML model, we consider the application of CNNs, the goal of which is sensing and prediction of the spectrum occupation (or availability), i.e., the actual creation of the mentioned common model of spectrum occupation in time, frequency, and location. We choose CNN ML because our previous research [2,13,16] proved that CNNs are a better choice for faded 5G signal detection than RNN, k-nearest neighbors, and Decision Trees algorithms, and do not need large and complex data sets, as they can find hidden patterns within data. The final ML models are created by the central FL node by intelligent averaging weights of the CNN and then shared for the use of SU. Consequently, the received CNN model from the FL server should be able to find intricate and complex dependencies in data with little or no preprocessing of the data.

The rest of the paper is organized as follows. In Section 2, we describe the considered system model and we define the scientific problem. In Section 3, we present the solution of the stated problem by applying federated learning iterative procedure, clustering, and ML-based spectrum occupancy model creation. In Section 4, we discuss verification of this solution via the computer simulation, we present detailed simulation assumptions and settings, and we discuss computer-simulation details. In Section 5, we derive the results of the said simulation. In Section 6, we derive the conclusions.

## 2. System Model and Problem Definition

In our work, we investigate the system where the PU’s activity is detected by means of a dedicated spectrum sensing system, where FL is applied to improve the detection of PU activity. The SS system consists of the centralized entity (FL server) and the set of randomly and densely deployed sensors (FL nodes), responsible for observation of the spectrum in their vicinity. The SU, which wants to utilize the unused spectrum fragments, communicates with the SS system to obtain the latest update on the prediction of the spectrum sensing occupancy depending on its current location. In our paper, we assume the correct operation of sensors and focus on solutions for the detection and prediction of resources. However, it should be emphasized that in the case of FL implementation, authentication of the participating FL nodes and information security must be supported. Examples of works on this subject can be found in [23,24].

As for the primary system, we focus on the detection of specific time-and-frequency patterns present in all contemporary and future radio communication systems. In particular, we analyze radio communication systems, where data are transmitted using so-called Resource Blocks (RBs), e.g., the 4G (Long-Term Evolution—Advanced, LTE-A) and 5G (New Radio, NR). They are defined in time (as a set of time slots) and frequency (as a set of adjacent Orthogonal Frequency Division Multiplexing (OFDM) subcarriers) as physical resources. Specifically, we consider the frequency division duplex (FDD) scheme and concentrate only on the downlink transmission. The SS system is aimed to detect occupied RBs to enable the transmission of SUs in a way that does not interfere with the PU’s signal. When analyzing the specific features of the transmitted DL signal (in terms of the periodicity, which can be revealed by the machine learning tools), one can observe that it is characterized by some occurring patterns. In the context of large time scales, the intensity of the traffic mainly fluctuates in time periodically, following daily changes in resource requirements. However, in the short time scale, one can notice that for the transmission, adjacent RBs (both in time and in frequency) are used, creating the resource block group (RBG) or their concatenation. This means that RBs typically occupy a solid frequency band. Another type of information that the learning mechanism can process is related to the wireless channel. Apart from the path loss (function of the distance between an SU’s or FL node’s receiver and a PU’s transmitter), the electromagnetic shadowing effect occurs and results in a specific relationship between the mean SNR value and location of a SU’s sensor. Finally, short-term frequency-selective Rayleigh fading (affecting a whole RB) and the Additive White Gaussian Noise (AWGN) are also present in the received signal.

**Figure 1:**
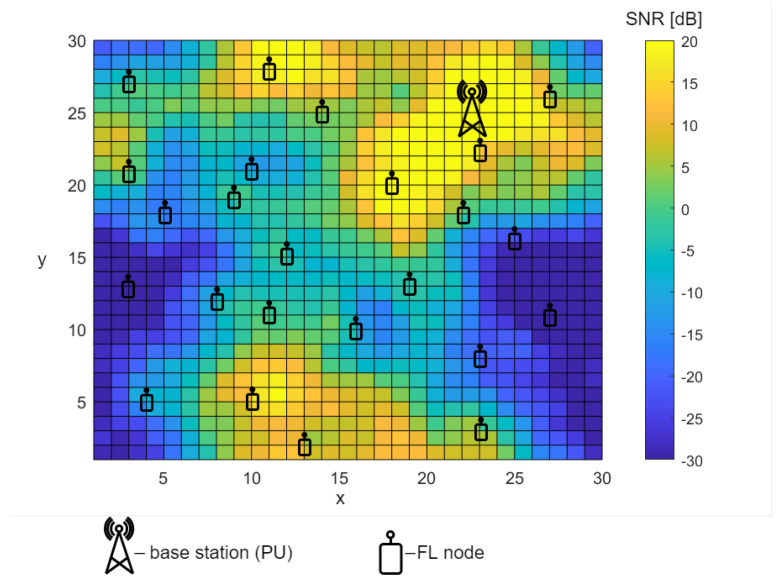
A map of mean SNR values in space, including FL nodes and a signal source—a PU.

In our system model, we consider one central FL server and a number *N* of sensors, which we will use as FL nodes located in a given area, as shown in Figure 1. The FL nodes are assumed to be able to perform ideal SS. This ability makes collection of labeled data possible. The FL nodes are distributed in the considered area so that the data collected by them are diverse and represent wireless channels with different propagation conditions. In Figure 1, one can observe an example SNR heat-map that represents the distribution of the true SNR value of the signal that originated from the PU. The variety of the SNR values, which change from −30 to 20 dB, is due to the terrain changes. We assume that the FL nodes can use the collected data to perform ML, namely CNN, which can extract information in an RB from the surrounding (in time and frequency) RBs that are not affected by the Rayleigh fading in a given moment and extrapolate decisions regarding occupancy of these RBs onto the currently faded RBs. This is the reason for the preference of CNN over simpler algorithms such as k-nearest neighbors or decision trees or the even more complex RNN. For K-nearest neighbors and decision tree algorithms to achieve the same results, additional data are needed for each RB. These data should consist of not only energy, time, and frequency characterizing a given RB, but also information on the energy of adjacent RBs. The more information on the energy of closer RBs, the better. To take into account the history of the signal, the energy value of RBs appearing a few time steps in the past should also be considered. The complexity of input features needed per RB grows when a less complex algorithm is chosen. The DL RNN algorithm can also be a good choice, and the results are comparable, but because CNN naturally works on two-dimensional data, we have decided to employ it for our FL-based time and frequency sensing.

Please note that we intentionally express the size of the area in normalized units (i.e., in distance units), as we wanted to make our analysis more generic. The appropriate granularity of the SNR map will need to be adjusted depending on the transmit power and true changes of the SNR value. Thanks to such an approach, our analysis can easily be adjusted to various 5G scenarios, mainly to both classic cellular mass-traffic or IoT applications.

When an FL node creates its own CNN model, it is well-fitted to the local data collected by this node. We postulate using the FL method in order to take advantage of multiple differently trained models and create new ML models that will be more general in their application but still specialized in SS for some specific channel conditions. We assume that the SUs do not participate directly in the FL process but rather take advantage of the ready CNN models generated as a result of the FL algorithm. This seems to be a good solution for SUs with limited computational resources, as SUs do not have to perform any ML training.

## 3. Federated Learning Algorithm for Spectrum Sensing

The FL method is a form of collaborative learning and relies on multiple models created by independent devices or nodes. The final model is created by merging the created models into one. The way the FL algorithm works can be centralized or decentralized [25]. The centralized FL means that there is one central server that orchestrates the process, i.e., manages FL nodes and creates a global ML model. In decentralized FL, there is no central server, and the participating nodes have to coordinate among themselves to create a global model. In this paper, we focus on the centralized version of the FL algorithm.

Below, we present and discuss the FL algorithm proposed as a solution for the 5G spectrum sensing problem. Our FL algorithm can be divided into four major steps that will repeat iteratively. In the first step, the FL server chooses (in case of the first iteration) or creates CNN models by merging models received from the FL nodes. The models picked for merging are delivered by FL nodes chosen in the clustering process. Here, we examine the k-means clustering algorithm [26]. The resulting global model for a given FL nodes cluster is created by applying *federated averaging*, which means that the weights of the resulting CNN model are equal to the average of CNN weights received from FL nodes from this cluster. We define the CNN model weights for a sensor *s* as $w_{s}^{i}$ (for the *i*-th iteration of the FL algorithm). The global model weights are then defined as the average.

(1) $$W_{c}^{i}=\frac{1}{N_{S_{c}^{i}}}\sum_{s}^{S_{c}^{i}}w_{s}^{i},$$

where $\forall s\in S_{c}^{i}$, and $S_{c}^{i}$ is a set of sensors that belong to a cluster *c* in algorithm iteration *i*$\forall c\in C^{i}$, where $C^{i}$ is a set of all clusters in an iteration *i*. $N_{S_{c}^{i}}$ is a number of sensors in a set of sensor from a cluster *c*.

With the global model ready, the algorithm proceeds to the second step, in which it selects the nodes to send the respective models to by applying clustering. The clusters are created based on the mean SNR values estimated and sent by the nodes. These values represent the current channel state at each node and are calculated over some time frame. Clustering is performed in each iteration of the algorithm, and its results may change over time.

In the third step, the FL server sends the created CNN models, common for each group of FL nodes according to the clustering results. Based on these models, the FL nodes collect their local data and modify the received CNN models by training. The training of the received CNN model is performed in each FL node by calculating weights $w^{i}$ that modify received weights $W^{i}$ to minimize loss function $l_{n}^{i}\left(X_{n}^{i},Y_{n}^{i};w\right)$, where $X_{n}^{i}$ defines collected signal information, and $Y_{n}^{i}$ is a classification target, which can represent a free or occupied spectrum. Therefore, we want to minimize the loss function, for each sensor *s*, such that

(2) $$\min_{w_{s}^{i}}L_{s}^{i}\left(w_{s}^{i}\right),\;\;\;\mathrm{where}\;\;\;L_{s}^{i}\left(w_{s}^{i}\right)=\frac{1}{N_{samples}}\sum_{n=1}^{N_{samples}}l_{n}^{i}\left(X_{n}^{i},Y_{n}^{i};w_{s}^{i}\right),$$

where $N_{samples}$ denotes the number of collected data information samples for which sensing decision is performed. After the training phase, in the last step, all of the modified CNN models are sent back to the server, where the global model for a given cluster is created, which concludes the last step of the algorithm. The loss of the cluster *c* is described as

(3) $$L_{c}^{i}\left(W_{c}^{i}\right)=\frac{1}{N_{S_{c}^{i}}}\sum_{s}^{S_{c}^{i}}L_{s}^{i}\left(w_{s}^{i}\right)$$

**Figure 2:**
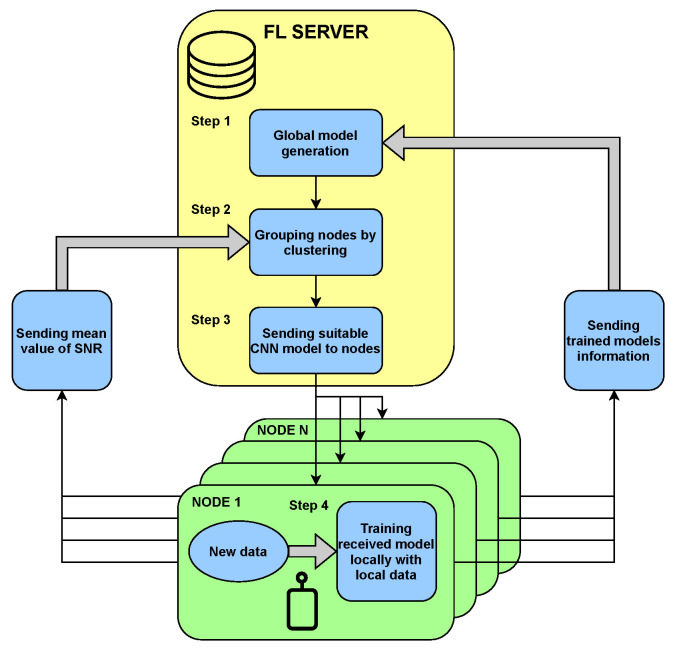
The algorithm of FL for SS.

The whole process repeats iteratively. The algorithm of FL in the form of a diagram is presented in Figure 2, and a pseudocode is as follows Algorithm 1.
**Algorithm 1** An algorithm of FL for SS.i←0I←N**while**i<I**do**    $C^{i}\leftarrow$ set of clusters (clustering sensors by mean SNR values)    **for each** $c\in C^{i}$ **do**        **for each** $s\in S_{c}^{i}$ **do**           **if** i==0 **then**               initialize $w_{s}^{i}$           **else**               $w_{s}^{i}\leftarrow W_{c}^{\left(i-1\right)}$           **end if**           calculate $w_{s}^{i}$ to minimize $L_{s}^{i}\left(w_{s}^{i}\right)$        **end for**        $W_{c}^{i}\leftarrow \frac{1}{N_{S_{c}^{i}}}\sum_{s}^{S_{c}^{i}}w_{s}^{i}$    **end for**    i←i+1**end while**

**Figure 3:**
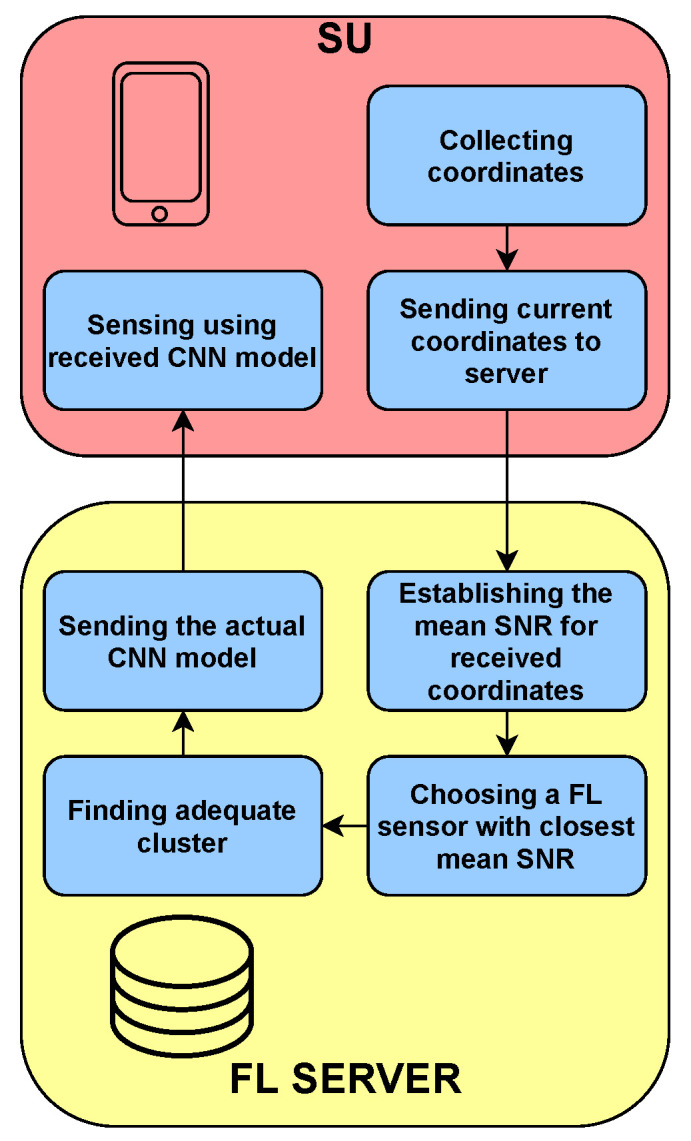
The algorithm of assigning the model to SU at a given location.

In our scenario, SU may appear anytime and anywhere in the considered area presented in Figure 1. If SU wants to perform sensing and gain information on the spectrum occupation, it should send its location to the FL server. The FL server is assumed to be aware of the mean SNR map of the area. Knowing the current location of the SU, the FL server can pick the best CNN model for this SU. Every time SU changes its location, it may request a new model for this location to ensure the best sensing performance. Moreover, even if the location of SU does not change, but the environmental conditions do, assigning a new model for a given location might be beneficial. The algorithm of SU’s service by the FL server is illustrated in Figure 3.

**Figure 4:**
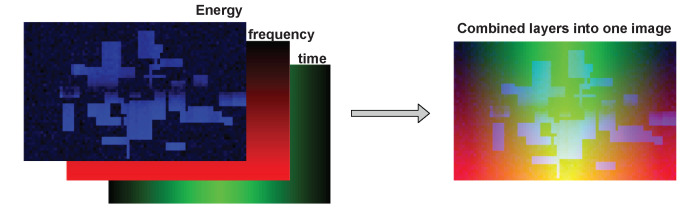
The CNN input data. The input image consists of three layers: energy values per RB, frequency indicator, and time indicator. The layers can be treated as RGB components.

After a new incoming SU receives the CNN model, it can feed it with its own collected data and perform sensing. The data collected by the user is of the same type as the data collected by the FL nodes. The main difference (and advantage) is that this new incoming SU does not need to acquire training data, as it already possesses a ready CNN model for SS. This means that it can simply measure energy values per RB and use them as CNN input. The data used during the training and testing phase consist of three values: the energy measured per RB and the frequency and the time denotation per RB (time-frequency coordinates of RB). These three values can be presented in the form of two-dimensional tables (pictures) and combined into three layers of one picture (or RGB components of an image). This representation makes it possible for CNN to process and find inner dependencies in the input data because the considered signal shows the correlation in both time and frequency. The additional two layers of frequency and time data emphasize those dependencies and make it easier for the CNN to find repeating patterns in time and frequencies that are potentially used more frequently. The number of rows of pixels in an image equals 50, and one pixel represents one resource block. The example of an input data image is presented in Figure 4, where three layers are combined into one CNN input picture.

## 4. Simulation Experiment

### Assumptions and Settings

In our experiment, we focused on sensing the 5G downlink signal. The tested signals have a 10 MHz bandwidth containing 50 RBs. Each of the RBs consists of 12 OFDM subcarriers. Each of the RBs lasts 0.5 ms and consists of 7 OFDM symbols. Full synchronization is assumed. The energy, frequency, and time information collection take place during the first OFDM symbol only, which leaves time for a decision on the occupancy of considered RB and the SU opportunistic use of the resources, i.e., transmission of signals, preferably orthogonal to the PU’s signal. A periodicity is present in the PU signal due to daily intensity oscillation. For simplification of the simulations, the oscillation occurs every 80 slots in time.

**Figure 5:**
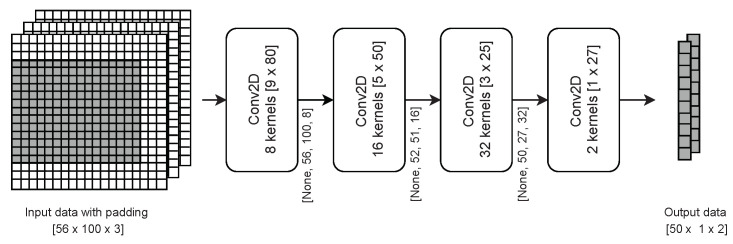
CNN algorithm model.

Regarding the selected CNN structure and algorithm, we have developed a four-layer CNN model. The input images consist of 50 pixels vertically and 100 pixels horizontally and have three layers. The activation function in all of the layers is the rectified linear activation function except for the last layer, where the softmax function is used as an activator. As an optimizer, the Adam optimizer is applied with a learning rate of 0.0001. The loss value is calculated using Sparse Categorical Crossentropy. As a result, the CNN returns two vectors of length 50. The vectors represent the occupancy probability of all RBs in a frequency band for a given moment. Each of the FL sensors creates such a CNN model and trains it on its collected data. CNN models of the same structure are created as a product of the FL procedure. The SU receives a ready CNN model, adequate for its location. The data collected by the SU is of the same type as training data. Figure 5 shows the details of the proposed CNN algorithm’s model.

The FL sensors collect data of length 800 slots in one iteration step. As the FL sensors are immobile, the fading effect is still in time. The environment is assumed to be quite dynamic, and in each FL algorithm iteration, the fading is different. This assumption equals a situation where, in each iteration, different FL sensors are picked, with a different fading channel, but with the same mean SNR as sensors from the previous iteration. It is assumed that the mean SNR values oscillate around values that are constant in time and characteristic for a given location. In our simulation, we chose 24 sensors locations randomly. The mean SNR values for those sensors are presented in Table 1.

In the simulation, k-means clustering is used to categorize FL sensors into groups of similar SNR values. We chose 8 as the number of clusters in our experiment. Table 2 presents an example of how the sensors were clustered in one of the FL algorithm iterations.

In the testing phase, we generated signals with different fading channels and combinations of SNR values. The SNR values are within the range [−24,−23,−22,…,16] dB. The tests are performed for all iterations from the range [1,2,3,…,30]. In each iteration, FL server chooses the best CNN model created by clustered FL sensors. The best model is chosen by the SUs’ and clusters’ mean SNR comparison.

We used Matlab software to simulate signals and the channel. The data for training and testing were also generated using Matlab. We applied the TensorFlow Python library, to create CNN models and use them in the FL algorithm.

## 5. Results

As a way to evaluate the obtained results, two probability measures were derived. First was a probability of detection $P_{d}$, which is defined as a probability of a decision function T(y) to correctly exceed the decision threshold λ, which is equivalent to saying that the signal is present. T(y) is defined by an SS algorithm of choice. For example, in the energy detection method, T(y) equals calculated signal energy. In energy detection, λ is equal to some energy value that is a limit value that determines the detection outcome. The value *y* is a set of input information, in the case of our experiment, y=[EV,t,f], where EV is an energy value and *t* and *f* are time and frequency information, respectively. Therefore, *y* is a set of features representing a given RB. On the other hand, the probability of false alarm $P_{\mathrm{fa}}$ is a probability of falsely assuming that the signal is present when in fact it is absent. Both probabilities can be described as follows:

(4) $$\begin{array}{cc} \; & P_{d}=\mathrm{Pr}\left\{T\left(y\right)>\lambda |H_{1}\right\},\\ \; & P_{\mathrm{fa}}=\mathrm{Pr}\left\{T\left(y\right)>\lambda |H_{0}\right\}, \end{array}$$

where $H_{1}$ is a hypothesis indicating that the signal is present and $H_{0}$ indicates that the signal is absent.

**Figure 6:**
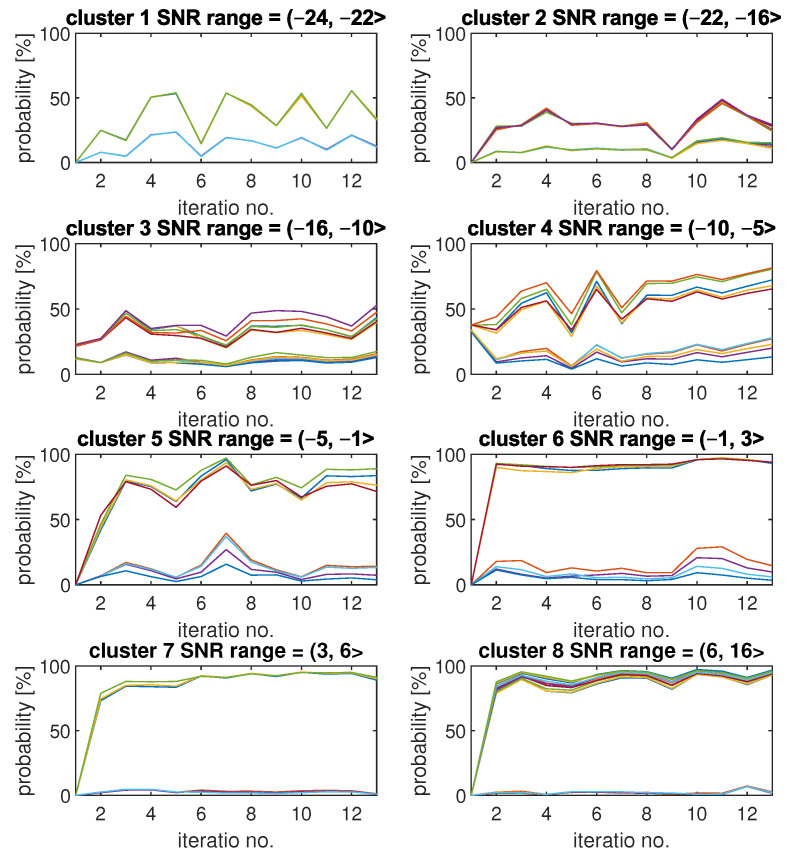
Exemplary changes of $P_{d}$ and $P_{\mathrm{fa}}$ for each FL algorithm iteration and for 8 different clusters.

As the first set of results, we present an example of how $P_{d}$ and $P_{\mathrm{fa}}$ of SU detection change with each iteration for each cluster. The results are included in Figure 6. Each plot was obtained by SU for all of the integer SNR values from a corresponding range included in the plot title.

Each plot is titled with the cluster index and SU SNR range that has been associated with this cluster. It can be observed that for clusters with relatively low values of SNR, the changes in results can be quite significant between each of the iterations. For example, $P_{d}$ results for cluster 1 tend to switch between values around 50% and around 15%, which correspond with higher and lower $P_{\mathrm{fa}}$ values. The high diversity of results for low SNR values can be explained by the high sensitivity of the CNN models for changes when the signal is so weak compared to noise. Clusters 7 and 8, which refer to high SNR values, show the smallest variety of results. These clusters were created by using CNN models with similar and relatively high detection quality, so changes in the fading channel between iteration do not have any significant impact on the results. Another interesting observation is that for the first two clusters, all $P_{d}$ curves are very similar to each other. The same is true for $P_{\mathrm{fa}}$ plots. Starting with the third cluster, up to the fifth, it is visible that those plots began to differ significantly, though the downward and upward trends of the curves remain the same. For clusters 6, 7, and 8, the results are similar again.

**Figure 7:**
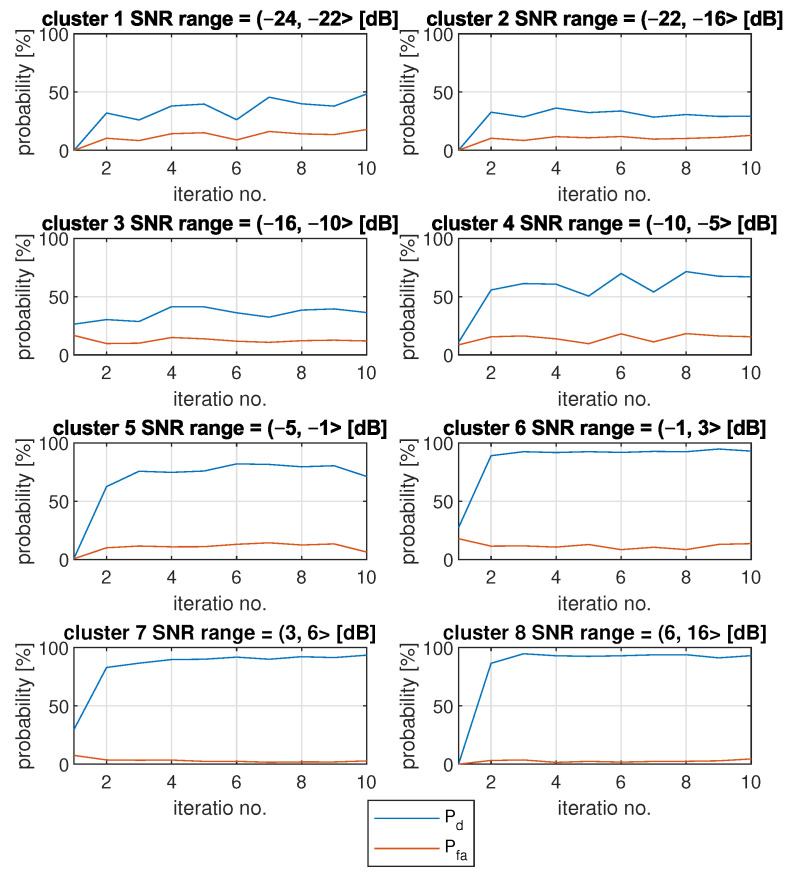
Mean changes of $P_{d}$ and $P_{\mathrm{fa}}$ for each FL algorithm iteration and for 8 different clusters.

Figure 6 presents one exemplary FL algorithm run. Figure 7 shows how the algorithm behaves on average. It includes mean results of $P_{d}$ and $P_{\mathrm{fa}}$ results for each cluster.

Each $P_{d}$ was calculated as an average of $P_{d}$ results of several simulations and all integer SNR values. The same goes for $P_{\mathrm{fa}}$ results. It can be observed that, on average, the results are quite steady after the second iteration. It is also visible that $P_{d}$ values do not necessarily grow with growing cluster numbers. For example, $P_{d}$ is around 40% for first cluster, and it reaches lower values (around 30%) for the second cluster.

**Figure 8:**
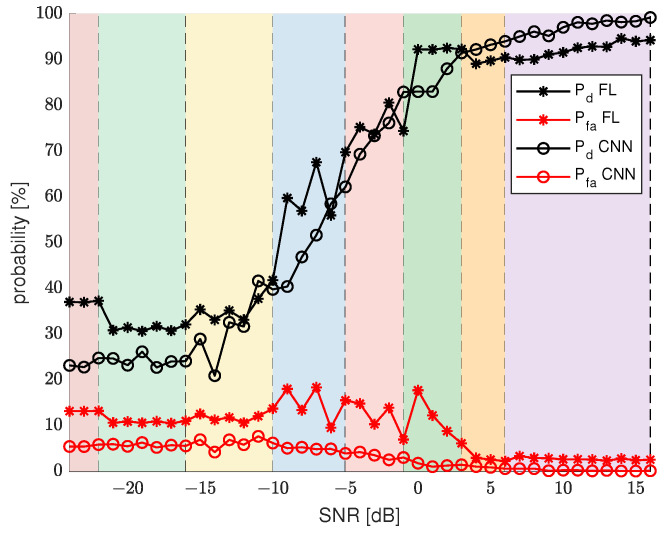
FL performance for different SNR values, for 8 clusters, compared with CNN results.

To correctly evaluate the final results, we calculated $P_{d}$ and $P_{\mathrm{fa}}$ for the same SU data, but employing separate CNNs that specialize in every SNR value from a considered range, and we used these results for comparison with FL algorithm results. Figure 8 presents $P_{d}$ and $P_{\mathrm{fa}}$ results for the final iteration of FL algorithm and $P_{d}$ and $P_{\mathrm{fa}}$ for basic CNN sensing. To improve the analysis of the final results, the plot was divided into separate sections marked by different colors. Each of the sections represents a range of SNR values that have been categorized into a different cluster.

The main conspicuous difference is that the CNN sensing probability plots are quite smooth and consistently change values with growing SNR without excessive fluctuations. On the other hand, the FL sensing results are diverse and depend a lot on a cluster and the range of SNR values of that cluster. For example, results for the first two clusters are quite stable. For clusters 3, 4, 5, and 6, results are quite various, which could already be seen by analyzing Figure 6. These clusters represent SNR ranges, for which $P_{d}$ results grow fast and $P_{\mathrm{fa}}$ results begin to decline. This causes more imbalance in the results, as there is a need for more specialized CNN models for each SNR value. For the last 3 clusters, the results are again more stable due to less variability in the expected results. The FL results show clearly that the clustering of the CNN models has an impact that is not negligible. The border SNR values between clusters are quite visible, as they often correlate with a sudden change in $P_{d}$ and $P_{\mathrm{fa}}$ results. Despite the variability in the results, the overall trend, similar to CNN sensing results, is maintained. In general, FL results improve for the same SNR values as CNN results. The one quite noticeable drawback of FL sensing is $P_{\mathrm{fa}}$ results for the middle clusters. Along with the dynamically changing $P_{d}$ results come higher values of $P_{\mathrm{fa}}$.

**Figure 9:**
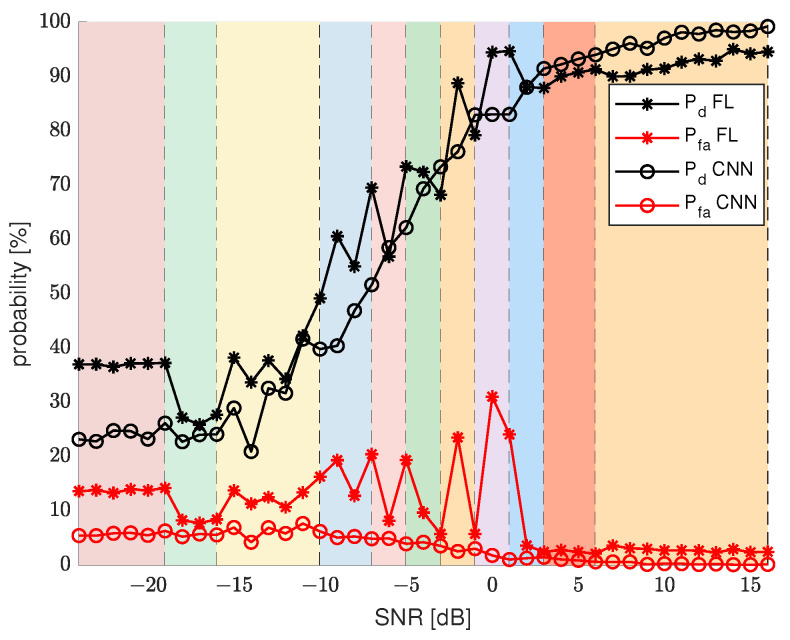
FL performance for different SNR values for 12 clusters compared with the CNN results.

The same type of results were generated additionally for 12 clusters. Figure 9 shows these results. Now, there are fewer FL sensors that contribute to model generation for each cluster. The consequences of that are visible especially for $P_{\mathrm{fa}}$ results for the middle SNR range. The fluctuations in the results are a bit more dynamic than in the results for eight clusters, but in general, the SS results are similar in both cases.

By analyzing the above figures, one can come into a conclusion, that in general the FL results, albeit a little variable, present a solid alternative to the more channel fitted SS ML models. The learning process does not need a lot of iterations on different channel conditions and is able to generate well working models using data collected by only a few sensors.

## 6. Discussion

In this paper, we discussed an application of FL for SS. It has been proven that the FL method makes it possible for SU to perform intelligent sensing without the need for extensive data collection and CNN model training. Although FL sensors are limited with their collected data to one mean SNR value, the common generated models are quite well prepared for SNR values from a given range. It has been shown that there is no need for many FL algorithm iterations. In fact, satisfactory results can be obtained in just a few iterations. Comparing our results with previously published FL sensing-related works, we focused on applying FL to take the burden of ML training off of SU and instead use FL sensors in the training process. Moreover, our algorithm employs FL sensors to create not one but multiple ML models that enable SS for diverse sensing conditions. Each of the models specializes in a separate SNR range.

To sum up, the main advantage of the proposed algorithm is offloading SU from ML training responsibility. The FL performance is comparable with the performance of the SS performed by CNN models specialized in a given SNR value. This means that data collected from a few FL sensors are enough to create averaged SS models that are enough to perform SS from the whole continuous range of SNR.

In future experiments, we will examine the impact of different numbers of clusters and different clustering algorithms on final results. Another interesting area of research that we will look into is decentralized FL.

## Figures and Tables

**Table 1:** Mean SNR [dB] of each FL sensor.

sensor 1	sensor 2	sensor 3	sensor 4	sensor 5	sensor 6
−30	−30	−22.9	−20.6	−17.04	−13
**sensor 7**	**sensor 8**	**sensor 9**	**sensor 10**	**sensor 11**	**sensor 12**
−11.8	−7.6	−7.3	−5.8	−3.4	−2.9
**sensor 13**	**sensor 14**	**sensor 15**	**sensor 16**	**sensor 17**	**sensor 18**
−1.8	−0.9	0.8	2.6	4.8	4.8
**sensor 19**	**sensor 20**	**sensor 21**	**sensor 22**	**sensor 23**	**sensor 24**
5.9	7	9.7	17.6	19.8	20

**Table 2:** Mean SNR (dB) of FL sensors in each cluster.

**cluster 1**	−30	−30	−22.9		
**cluster 2**	−20.6	−17.04			
**cluster 3**	−13	−11.8			
**cluster 4**	−7.6	−7.3	−5.8		
**cluster 5**	−3.4	−2.9	−1.8	−0.9	
**cluster 6**	0.8	2.6			
**cluster 7**	4.8	4.8	5.9		
**cluster 8**	7	9.7	17.6	19.8	20

## Data Availability

Generated data by authors: https://doi.org/10.5281/zenodo.5805530 (accessed on 22 November 2021).

## Abbreviations

The following abbreviations are used in this manuscript:

SS	Spectrum Sensing
PU	Primary User
SU	Secondary User
ML	Machine Learning
DL	Deep Learning
RNN	Recurrent Neural Network
SVR	Support Vector Regression
CNN	Convolutional Neural Network
KNN	K-Nearest Neighbors
RF	Random Forest
FL	Federated Learning
SAS	Spectrum Access System
CBRS	Citizen Broadband Radio Service
CSS	Cooperative Spectrum Sensing
SNR	Signal-to-Noise Ratio
RB	Resource Block
LTE-A	Long-Term Evolution-Advanced
NR	New Radio
OFDM	Orthogonal Frequency Division Multiplexing
FDD	Frequency Division Duplex
RBG	Resource Block Group
AWGN	Additive White Gaussian Noise
